# Late Radiation–Related Toxicities in Patients Treated for Early-Stage Cervical Carcinoma by Surgery and Adjuvant Radiotherapy: A Retrospective Imaging Study

**DOI:** 10.3389/pore.2021.1609915

**Published:** 2021-09-28

**Authors:** Katarina Nadova, Miroslava Burghardtova, Klara Fejfarova, Klaudia Reginacova, Hana Malikova

**Affiliations:** ^1^ Radiology Department, Third Faculty of Medicine, Charles University, Faculty Hospital Kralovske Vinohrady, Prague, Czechia; ^2^ Second Faculty of Medicine, Charles University, Prague, Czechia; ^3^ Oncology Department, Third Faculty of Medicine, Charles University, Faculty Hospital Kralovske Vinohrady, Prague, Czechia; ^4^ Institute of Anatomy, Second Medical Faculty, Charles University, Prague, Czechia

**Keywords:** cancer, toxicity, radiation-induced tumor, complication, bone, comorbidity

## Abstract

Surgical treatment is preferred therapy of early-stage cervical carcinoma. In the risk of cancer recurrence surgery is often followed by adjuvant radiotherapy. In our retrospective study we aimed at identifying late (≥6 months) and very late (≥5 years) radiation adverse effects on imaging scans as CT, PET/CT and MRI in patients who underwent successful treatment for cervical carcinoma by radical surgery combined with radiotherapy ± chemotherapy. We correlated imaging results with clinical manifestations. We selected young and middle-aged patients with long life expectancy, as late radiation-related toxicities may significantly affect their quality of life. Patients were selected from those who were primary diagnosed and treated between the years 1987–2011 and regularly visited our Oncology department in years 2011–2012. Following inclusion criteria were applied: age ≤55 years at diagnosis, clinical follow-up ≥5 years and at least one tomography scan ≥3 years after finished treatment. One hundred and three subjects were reviewed: 73 patients met all inclusion criteria, while 30 patients fulfilled the inclusion criteria except for available tomography scan ≥3 years after therapy. The mean imaging follow-up was 11.2 ± 7.6 years and the mean clinical follow-up was 15.0 ± 6.9 years. In 20 (27%) subjects 27 cases grade I radiation-related toxicities were found; 9 (33%) of those 27 cases were clinically silent. In 14 (19%) females only grade I toxicities were observed. Grade III-IV toxicities were found in 5 (6.8%) subjects. No grade V toxicities were observed. We concluded that severe late side effects caused by radiotherapy were exceedingly rare in females successfully treated for early-stage cervical carcinoma, only 1 bilateral osteonecrosis, 2 cases of ileus, and 2 potentially radiation-induced tumors were found. The majority of radiation-related comorbidities found on imaging scans were clinically silent.

## Introduction

Cervical carcinoma is serious health problem worldwide, is the fourth most common cancer in females and the seventh most common cancer overall^1,2^. Despite of current progress in diagnosis and treatment, cervical carcinoma still remains the most common cause of tumor-related death in women under the age of 35 years^1,2^. However, there is evidence of declining incidence in developed countries in recent years due to cervical screening programs and HPV vaccination efforts^1,2^.

The International Federation of Gynecology and Obstetrics (FIGO) system is commonly used for cervical carcinoma staging^2^. Radical surgery is commonly used for the treatment of early stages up to IB2 and IIA1. In younger patients with early-stage disease, fertility-sparing surgery such as conization and trachelectomy is considered. Postoperative adjuvant radiotherapy (RT) is required in cases with a high risk of tumor recurrence such as: tumor larger than 4 cm, lymphovascular invasion, invasion of the outer 1/3 of the cervical stroma, positive surgical margins or invasion of the parametria and positive lymph nodes^2,3^. For advanced stages, IIB and higher, chemotherapy and combined external beam radiation therapy (EBRT) and intracavitary brachytherapy (BRT) are the main treatment options^2^.

According to FIGO guidelines the pelvic MRI is the best method for radiological evaluation of primary cervical carcinoma ≥10 mm in size and for cancer staging or restaging^2,4,5^. The role of PET/CT has recently been discussed, especially for lymph nodes assessment^2^.

It is well known that combined radiation therapy (EBRT and BRT) is associated with broad spectrum of comorbidities. Moreover, they may be additionally affected by previous surgical treatment and chemotherapy. Radiation-related comorbidities that appear within 6 months after RT are considered acute, while radiation-related comorbidities that appear later than 6 months after RT are considered late^6^. The most radiosensitive and vulnerable tissues at the site of RT are tissues with high proliferative activities, which include bone marrow, both the small and large bowel and the urinary bladder. Patients may suffer from radiation-related proctocolitis, enteritis and cystitis, not uncommon are ileus, bowel perforation, fistulas, enteral/colic and ureteral strictures and even hydronephrosis. Bone marrow fat degeneration is unavoidable process. However, patients may suffer from osteonecrosis and bone insufficiency fractures^6^. Radiation-induced tumors must be also considered in the spectrum of very late radiation-related comorbidities^7^.

Many studies have provided data about radiation-induced toxicities related to radical RT/chemoradiotherapy for advanced cervical cancer^8^. Acute radiation adverse effects are well documented. However, there is lack of data in the literature that document late and especially very late radiation toxicities ≥5 years^8^. In our previous study, we showed very late radiation-related comorbidities after radical RT for advanced cervical cancer and suggested that if successfully treated females present with abdominal/pelvic complaints more than 5 years after RT, it is highly probable that tomography scans will show late radiation-related side effects, which probably influence the quality of patient’s lives^9^. Few studies have reported late radiation-related data after adjuvant RT, which uses substantially lower radiation doses than radical combined RT^10^. We therefore focused on females treated for early-stage cervical cancer by radical surgery and adjuvant combined RT. In our retrospective study we aimed at identifying late (≥6 months) and very late (≥5 years) radiation adverse effects on imaging scans as CT, PET/CT and MRI in patients who underwent successful treatment for cervical carcinoma by radical surgery combined with RT ± chemotherapy. We were interested in young and middle-aged patients with long life expectancy as late radiation-related comorbidities could seriously influence the quality of their lives and their future well-being.

## Materials and Methods

This retrospective study was initiated in January 2018. We selected all female patients with a history of early-stage cervical cancer treated by radical surgery (hysterectomy, adnexectomy, appendectomy and pelvic lymph nodes dissection) and adjuvant RT ± chemotherapy that visited our Oncology department between years 2011 and 2012. Some patients underwent surgery ± RT at other institutions; however, all were clinically followed at our hospital. Patients that were treated at our institution received following radiation doses: BRT usually 2 (or 3) x 5 Gy, pelvic EBRT 45–48.6 Gy (upper edge of L5). The inclusion criteria for our study group were as follows:

• Patients in the age ≤55 years at the time of cancer diagnosis.
• Five years survival since therapy.
• One tomography scans as CT, PET/CT or MRI ≥3 years after the end of the cancer treatment. We excluded all females who were observed by ultrasonography and chest X-rays.
• At least 5 years of complete clinical follow-up with available clinical data.

On imaging scans we assessed:

• Signs of chronic radiation-related cystitis with fibrotization of the bladder wall with diffuse or local irregularities or chronic bladder wall edema.
• Signs of hydroureter ± hydronephrosis due to distal ureteral strictures.
• Signs of bowel wall thickening, stranding and bowel wall edema as a result of chronic enteritis and proctocolitis. Signs of their complications as bowel perforation ± pneumoperitoneum. Signs of ileus due to bowel strictures.
• Bone complications such as osteonecrosis and insufficiency fractures (presence of fatty bone marrow replacement was considered a common finding, not a radiation-related comorbidity).
• Possible radiation-induced secondary malignancies.

Only diagnosis that were depicted ≥6 months after finishing RT were evaluated as the late radiation-related side-effects.

For the grading of radiation-related adverse effects we used the common terminology criteria for adverse events version 5.0 (Ref. 11). For simplified criteria of the grading system see also Table 1.

**TABLE 1:** Simplified criteria of the grading system for the radiation-related adverse events.

Grade		Description of adverse events
I.	Mild	• Asymptomatic or mild symptoms
		• Clinical or diagnostic observation only
		• Treatment is not indicated
II.	Moderate	• Minimal or local events
		• Conservative treatment indicated
		• Limiting age appropriate instrumental activities for daily living
III.	Severe	• Disabling events
		• Medically significant but not immediately life-threatening
		• Hospitalization or prolongation of hospitalization indicated
		• Limiting self-care activity of daily life
IV.	Life-threatening	• Life-threatening events
		• Urgent treatment is necessary
V.	Death	• Death related due to radiation-related adverse effect

Our retrospective study was conducted according to Declaration of Helsinki. The study was approved by the local Ethics committee; the informed consent was waived by the Ethics committee due to a retrospective design of the study.

## Results

### Patient Selection

All available medical records of 103 females treated for early-stage cervical cancer that survived more than 5 years from diagnosis were reviewed; 73 patients met all inclusion criteria, while 30 patients were followed by ultrasonography and chest X-ray and were excluded from the radiology imaging study. All patients underwent radical surgery followed by combined RT (EBRT and BRT) ± chemotherapy. No significant pretreatment comorbidities were found in their medical records. Patient selection data are shown in Table 2.

**TABLE 2:** Patient selection and demographic data.

	Included pts with available imaging and clinical data	Pts with available clinical data with no imaging
No of pts	73	30
Age	40.0 ± 7.8 years	33.5 ± 6.5 years
The last available imaging scan (CT, PET/CT, MRI)	11.2 ± 7.6 years	-
Time of clinical follow-up	15.0 ± 6.9 years	22.0 ± 8.8 years
^a^Number of females who survived from RT to 2018	65 pts	30 pts
^a^Number of females that did not survive to 2018	8 pts (3 late cancer recurrence, 3 duplicate cancer)	0 pts
Time between RT to pts death	16.1 ± 6.2 years	
Chemotherapy	27 pts	2 pts (cisplatin)
	(19 pts cisplatin	
	3 pts combination mitomycine and vincristine	
	5 pts no data available)	
Histological origin of cervical cancer	57 epidermoid carcinomas	21 epidermoid carcinomas
	13 adenocarcinomas	9 adenocarcinomas
	2 clear cell carcinomas	
	1 carcinosarcoma	

a 2018 = the year of the initiation of the study. (CT, computerized tomography; No, number; PET/CT, positron emission tomography/computerized tomography; pts, patients; RT, radiotherapy.)

### Late Radiation-Related Comorbidities

**FIGURE 1:**
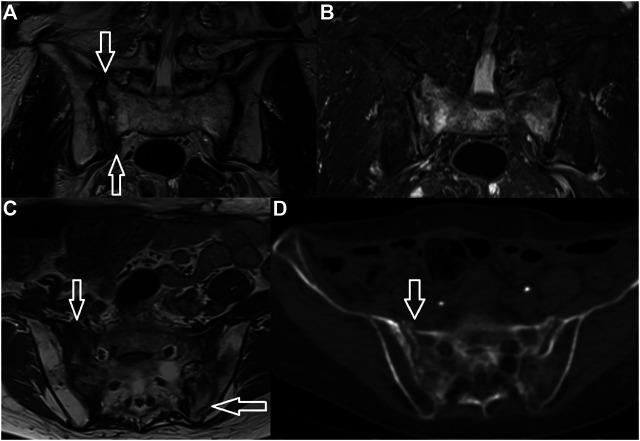
Insufficiency fracture. H-shaped sacral fracture is well-visible both on MRI and CT (arrows) (**A**–TSE T2 WI, **B**–TSE T2 FAT SAT, **C**–TSE T1 WI, **D**–CT).

**FIGURE 2:**
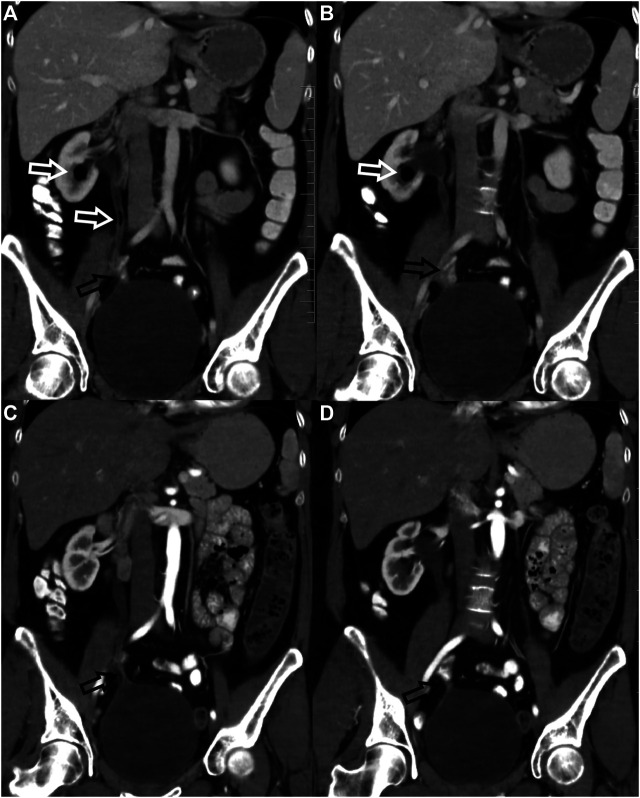
Clinically-silent uretero-enteral fistula. Uretero-enteral fistula was found incidentally 7 years after RT in a patient after right ureter-to-bladder reimplantation due to early postoperative stenosis. The patient did not suffer from any clinical manifestations, urinalysis was normal. CT revealed air bubbles in the right ureter and renal calyx (white arrows–**(A,B)** due to the uretero-enteral fistula located in the vicinity of ureter reimplantation. There was no evidence of patent fistula on CT 1 year later **(C,D)**, the site of ureter reimplantation was clearly visualized (arrows).

Most radiation-related comorbidities were minor (grade I), 27 cases of grade I toxicities in 20 (27%) subjects were found. However, 9 (33%) cases of 27 grade I toxicities were clinically silent and were apparent only on CT, PET/CT or MRI. In 14 (19%) females were only grade I toxicities found. In 2 subjects (2.7%), asymptomatic bone comorbidities (insufficiency fractures and pelvic osteonecrosis) were diagnosed, see also Figure 1. The most interesting case in this group of patients was a case of an entero-ureteral fistula, found in a 40-year-old woman who underwent radical surgery at the age of 33. Cervical cancer was diagnosed by gynecology examinations, vaginal ultrasound and cervical biopsy. Definitive histology after radical surgery revealed cervical adenocarcinoma without microscopic invasion of the parametria; in 4 lymph nodes metastasis were found. In time of the diagnosis both vaginal and abdominal ultrasound and also CT scans done before adjuvant RT did not reveal hydronephrosis or the urinary bladder infiltration. She underwent adjuvant combined RT and chemotherapy. Two months later, ultrasound revealed right-sided hydronephrosis, which was treated by ureteral pig-tail placement. Two years later she underwent surgery with ureteral reimplantation. She was followed by CT and PET/CT. Five years after ureteral reimplantation, an entero-ureteral fistula was incidentally found on CT; the patient did not suffer from any clinical manifestations and urinalysis was normal. Follow-up CT 1 year later showed normal findings; the fistula had closed spontaneously. For more details see also Figure 2.

No grade II radiation-related comorbidities were found.

**FIGURE 3:**
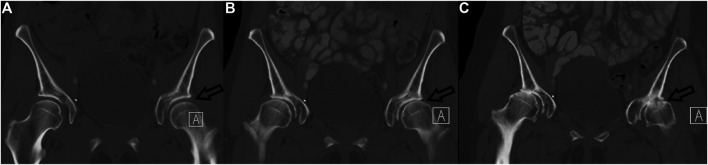
Radiation-related bilateral femoral head necrosis. Three coronal CT images of hip over 9 years follow-up are presented (2009–**A**, 2016–**B**, 2018–**C**). Note rapid bilateral hip osteoarthritis development, from normal **(A)** to bilateral femoral head necrosis, which is better seen on the left (**C**–arrow).

Grade III comorbidities were found in 3 (4.1%) subjects, all of which were solitary complications:

• Two cases of ureteral strictures with hydronephrosis were found 6 and 12 months after RT.
• One case of bilateral osteonecrosis of the femoral heads was found and total bilateral femoral endoprosthesis was indicated (Figure 3).

Grade IV comorbidities were found in 2 (2.7%) subjects. Both of them suffered from chronic enteritis with strictures and ileus manifested 14 months and 7 years after RT, respectively. Both females underwent surgery with bowel resection; diagnosis of radiation enteritis was proven histologically.

In addition, we speculated about 2 (2.7%) cases of potential radiation-induced tumors at the site of RT. Both women underwent treatment for cervical cancer in the age of 33. First one suffered from cervical adenocarcinoma, 18 years after RT epidermoid carcinoma in the vaginal vault was diagnosed. The second patient was treated for epidermoid cervical cancer and 22 years after therapy urothelial carcinoma of the urinary bladder was found. Thus both tumors were different histological origin than primary cervical cancer. Both tumors were treated by surgery and histology except of tumor infiltration revealed also chronic radiation-related changes in the surrounding tissue.

We did not observe any grade V radiation-related comorbidities. All collected data are summarized in Table 3.

**TABLE 3:** Late radiation-related toxicities (≥6 months after adjuvant radiotherapy).

Comorbidities		Grade	No of cases	Only imaging findings (no clinical manifestation)	Time of presentation on imaging (After RT)
Urological	Mild cystitis	I	10	3	^a^NA
			(13.7%)		
	Moderate - severe cystitis	II	0	0	0
	Ureteral strictures and hydronephrosis	III	2	0	Min. 6 months, max 12 months
			(2.7%)		
GIT	Mild Proctocolitis/enteritis	I	14	3	^a^NA
			(19.2%)		
	Moderate proctocolitis/enteritis	II	0	0	0
	Ileus	IV	2	0	Min. 14 months, max. 7 years
			(2.7%)		
Fistulas	Entero-ureteral	I	1 (1.4%)	1	7 years
Bone toxicities	Insufficiency fractures	I	2 (2.7%)	2	Min. 1.5 years, max. 26 years
	Osteonecrosis surgery or other invasive therapy required	III	1	0	11 years
			(1.4%)		
Radiation-induced (probable) tumors		-	2 (2.7%)	0	Min 18 years, max. 22 years

a NA, not assessed; signs were apparent or clinically present during the entire follow-up period. (GIT, gastrointestinal tract; No, number; RT, radiotherapy.)

Moreover, we calculated the rate of all serious toxicities ≥ grade III in entire group of patients with available complete clinical data (103 females) that manifested clinically; those data are summarized in Table 4.

**TABLE 4:** Late grade III-V radiation-related toxicities in all patients with complete clinical data (including patients who were excluded from radiology imaging study).

Grade III-V comorbidities	All reviewed patients with clinical data
	No = 103
No of all affected pts (radiation-induced tumor included)	7 (6.7%) of 103 pts
Severe urinary bladder toxicities	0 of 103 (0%)
Fibrous ureteral strictures	2 of 103 (1.9%)
Ileus/bowel perforation	2 of 103 (1.9%)
Various fistula formations	0 of 103 (0%)
Severe bone complications	1 of 103 (1.0%)
Radiation-induced tumors (possible)	2 of 103 (1.9%)

(No, number; pts, patients; RT, radiotherapy.)

## Discussion

In our present retrospective study, we evaluated late and very late radiation-related comorbidities after adjuvant RT in young and middle-aged females who underwent radical surgery for early-stage cervical carcinoma and survived more than 5 years. The mean clinical follow up was roughly 15 years, and the mean imaging follow-up was approximately 11 years. We focused on younger patients with long life expectancy, we wondered if potential radiation-related comorbidities could seriously influence the quality of their lives and overall well-being. We assessed the rate of radiation-related comorbidities on tomography scans, including clinically silent cases and cases with minimal clinical manifestations.

We did not find any grade V toxicities. Grade III toxicities occurred in 3 (4.1%) subjects; all were solitary and manifested minimally 6 months and maximally 11 years after RT. We found 2 (2.7%) cases of grade IV radiation-related side effects, both were ileus due to chronic radiation enteritis and required urgent surgery, one of them occurred 14 months after RT the second one 7 years after RT. We found only 2 cases of late toxicities grade III-IV more than 14 months after radiotherapy; one case of ileus (7 years after RT) and one case of bilateral femoral head osteonecrosis (11 years after RT). Moreover, 2 potentially radiation-induced tumors were discovered 18 and 22 years after RT. If we counted all subjects with complete clinical data who were reviewed, thus also subjects who were excluded due to lack of available imaging, the rate of late grade III-IV toxicities would be 3.8% (possible radiation-related tumors excluded). Our data suggest that the life of patients treated by surgery followed by combined adjuvant RT is not seriously affected by late radiation-related toxicities.

Comparison of our results with other studies is not straightforward. First, studies that provide late toxicity data due to adjuvant RT for early-stage cervical cancer are sporadic. Second, the follow-up period of our patients was extraordinary long. Most other studies have reported data from relatively shorter clinical follow-up periods, typically about 3–5 years. This approach is in concordance with FIGO recommendations; according to FIGO guidelines patients should be followed for 5 years, after which patients should return to common screening programs. Imaging (CT, MRI and PET/CT) is not a standard recommendation; patients are followed mostly by chest X-ray and ultrasound^2^. However, some data for comparison are available in the literature. Yuce Sari et al. reported serious late radiation-related toxicities in 7% of patients^10^. They included 113 females who underwent adjuvant RT, their median follow-up was 58–67 months and imaging was generally not provided^10^. On the contrary, no serious late toxicity after adjuvant RT was reported in a study by Yu et al.^12^. Interesting findings are found in a retrospective study by Fejardo et al.^13^. They studied 292 females treated between 1970 and 2007. All patients received EBRT 40–46 Gy and BRT 10–14 Gy. The incidence of 5 and 10 years cumulative risk of late complications was 11 and 13%, respectively^13^. They also reported increased risk for grade III or IV toxicities when chemotherapy was added, rising from 2 to 7%; however, that effect was apparent only in follow-up periods longer than 4 years^13^. Unfortunately, we do not have sufficient data available for comparison; only 27 of 73 patients in the present study received additional chemotherapy.

Radiation-induced tumors are considered very late radiation-related comorbidities^14^. We speculated about 2 cases of possible radiation-induced secondary tumors, one urothelial and one vaginal carcinoma. Both fulfilled Cahan’s criteria for radiation-induced tumors^15^. They were found in the field of RT, in the organs which were not affected before treatment, with a sufficient latent period (from 18 to 22 years) and histological origin of the induced tumor were clearly different from primary treated cancer^15^. According to literature data organs receiving low-dose radiation tend to development of carcinomas as secondary induced tumors^16^. Opposed to sarcomas affected more often organs that were irradiated by high doses^16^. This is in accordance with our results; we did not find any secondary sarcoma, but two probable cases of secondary carcinoma. However, in our previous study we reported one case of radiation-related sarcoma in the iliac bone after radical RT for cervical cancer^9^. Yuce Sari et al. reported one case of possible osteosarcoma in the iliac bone as a late complication of adjuvant RT. Hall and Wuu calculated the relative risk of radiation-induced malignancy after RT for cervical carcinoma and came to following results: relative risk for bladder carcinoma is 4.5, for vaginal cancer 2.7 and for bone tumors 1.3^16^.

Skeletal complications are also included in late radiation-induced toxicities. Fat degeneration of the bone marrow is expected pathophysiological process which is always present at the site of the radiation field^17,18,19^. Skeletal toxicities are not surprising consequence of pelvic cancer radiation they are results of decreased bone mineralization and elastic resistance and even radiation-related necrosis^19^. Some studies reported increased bone toxicity after concurrent chemoradiotherapy for gynecological cancers^12,20^. We found osteonecrosis or insufficiency fractures in roughly 4% of patients that underwent adjuvant RT, and just 1 case was assessed as grade III. In the study of Schmeler et al. pelvic fractures were found in 9.7% of females who underwent radical RT; however, 55% of them were asymptomatic^20^. Minority of patients, 38%, were diagnosed in the first year after radical RT^20^. Kwon et al. conducted retrospective MRI study and evaluated 5-years cumulative prevalence of insufficiency fractures after radical RT for cervical cancer^17^. They concluded 5-years cumulative prevalence was 45.2% and the median time of their diagnosis was 16.9 months^17^. According to above mentioned data, late bone toxicities may be underdiagnosed as a substantial amount are clinically silent^17,20^.

With the exception of asymptomatic bone toxicity, we found several cases of mild cystitis, enteritis and proctocolitis, which were apparent only on imaging (CT/MRI). These findings are not surprising and have been described previously^6^. Moreover, we incidentally found a case of the uretero-enteral fistula which was clinically silent and closed spontaneously (Figure 2).

In our previous study we evaluated late radiation-related comorbidities in patients successfully treated by radical RT for advanced cervical cancer^9^. We concluded that in case those women present with abdominal/pelvic complaints more than 5 years after RT, it is highly probable that tomography scans will show late radiation-related side effects, which probably influence the quality of their lives^9^. Opposed to in our present study we concluded that adjuvant RT does not affect future health of treated women. We explain those results not only by the higher dose of applied RT but also the extend of the RT field. In case of radical RT following doses are usually applied: BRT 6.5–7 Gy (4 times), pelvis EBRT 45 Gy (with the upper edge of L5); however, in case of the common iliac lymph node/s affection, the upper edge of EBRT is higher at the level of L4. Moreover, in case of the paraaortic lymph nodes affection additional EBRT of paraaortic lymph nodes is applied with simultaneous integrated boost only to affected lymph node to 55 Gy. Moreover, radical RT is often applied in combination with chemotherapy that may contribute to severity of radiation-related effects^12,13,20^.

The present study has several important limitations. It is a retrospective single center study. However, a prospective design with such a long follow-up period (mean 15 years) would be very difficult. It is necessary to admit that the most important limitation is selection bias. Our patients were highly selected from those who were successfully treated and survived 5 years from initiating therapy and underwent at least one imaging scan more than 3 years from initiating therapy. The diagnostic tomography scans were not indicated regularly, patients were sent to scans mostly due to unspecific complaints or in subacute, acute or emergency situations. This approach is in a concordance with the 2018 FIGO report^2^. According to the 2018 FIGO report routine imaging is not indicated in patient follow-up and patients should be clinically followed-up no more than 5 years and then should be returned to gynecology screening programs^2^. This approach may have led to an overestimation of some cases of toxicities, as many patients without clinical manifestations or with minimal clinical complaints may have been lost to follow-up and only patients with clinical manifestations were followed by an oncologist and/or a gynecologist. Moreover, our institution took care of patients that were originally treated in other institutions by different radiation-based technologies, and using different planning software; therefore, the applied doses were not available in some patients. We also must admit that previous radical surgery may contribute to the development of late radiation-related comorbidities.

### Conclusion

Our results suggest that the quality of life of young and middle-aged women with early-stage cervical carcinoma successfully treated by surgery followed by adjuvant RT is not generally affected by late and very late radiation-related toxicities. Severe late radiation-related comorbidities in these patients are very rare.

## Data Availability

The original contributions presented in the study are included in the article/Supplementary Material, further inquiries can be directed to the corresponding author.

## Glossary

BRT: brachytherapy
CT: computerized tomography
DSA: digital subtraction angiography
EBRT: external beam radiotherapy
FIGO: International Federation of Gynecology and Obstetrics;
HPV: human papilloma virus
L4: four lumbar spine vertebrae
L5: five lumbar spine vertebrae
MRI: magnetic resonance imaging
PET: positron emission tomography
RT: radiotherapy.
